# Cancer Vaccines in Genitourinary Malignancies: Current Advances and Future Directions

**DOI:** 10.3390/vaccines14060497

**Published:** 2026-06-01

**Authors:** Haider Altay, Ibrahim Al-Hashimi, Josh Matthews, Grace DeAlessandro, Ghanshyam H. Ghelani

**Affiliations:** Hematology and Medical Oncology, The University of Texas Health Science Center at Tyler, Tyler, TX 75708, USA; ibrahim.al-hashimi@uttyler.edu (I.A.-H.); josh.matthews@uttyler.edu (J.M.); gdealessandro@patriots.uttyler.edu (G.D.); ghanshyam.ghelani@uttyler.edu (G.H.G.)

**Keywords:** genitourinary cancers, cancer vaccines, immunotherapy, neoantigens, tumor microenvironment, prostate cancer, renal cell carcinoma, urothelial carcinoma

## Abstract

Therapeutic cancer vaccines are a promising immunotherapy approach in genitourinary (GU) cancers, designed to stimulate antitumor immune responses through antigen-specific T-cell activation. Although agents such as bacillus Calmette–Guérin in bladder cancer and sipuleucel-T in prostate cancer have shown success, vaccine monotherapy has generally produced limited clinical benefit due to tumor heterogeneity, poor immune infiltration, and immunosuppressive tumor microenvironments. Multiple vaccine platforms have demonstrated safety and immunogenicity in prostate, renal cell, and urothelial cancers, but efficacy remains modest. Current strategies focus on multi-antigen targeting, improved antigen presentation, and combination therapies with immune checkpoint inhibitors, radiotherapy, and targeted agents to enhance antitumor activity. Advances in personalized vaccine design and delivery systems are driving progress, though challenges such as manufacturing complexity, cost, and biomarker development remain. Ongoing translational and clinical research will be critical to improving the effectiveness of vaccine-based immunotherapy in GU malignancies.

## 1. Introduction

Cancer vaccines are a promising immunotherapeutic approach that stimulates host antitumor immunity through antigen-specific T-cell activation and antigen cascade effects. Unlike chemotherapy, therapeutic vaccines primarily target the immune system. Vaccine antigens are presented to dendritic cells, which activate and expand tumor-specific T cells capable of mediating antitumor responses.

Vaccine efficacy may improve when combined with chemotherapy, radiation therapy, immune checkpoint inhibitors, or tyrosine kinase inhibitors, potentially producing synergistic effects. Advances in cancer immunology and genomic sequencing continue to support vaccine development.

Therapeutic vaccines have shown the greatest benefit in settings of low tumor burden or earlier-stage disease progression [1]. Genitourinary (GU) malignancies have seen major advances in immunotherapy, including intravesical bacillus Calmette–Guérin (BCG) for superficial bladder cancer in 1976 [2], high-dose interleukin-2 for metastatic renal cell carcinoma in 1992 [3], and interferon plus bevaciz for metastatic renal cell carcinoma in 2010 [4]. Immune checkpoint inhibitors further expanded treatment options, with nivolumab approved for advanced renal cancer in 2015 based on CheckMate 025 [5] and atezolizumab approved for bladder cancer in 2016 [6]. Sipuleucel-T was approved for castrate-resistant prostate cancer in 2016 [7,8].

This article reviews cancer vaccine platforms and their therapeutic potential in GU malignancies.

## 2. Immunologic Barriers in Genitourinary Cancers Relevant to Vaccine Development

### 2.1. Tumor Antigen Selection in Genitourinary Malignancies

Shared tumor antigens remain central to therapeutic vaccine development in genitourinary (GU) cancers. In prostate cancer, prostate-specific antigen (PSA) has been widely studied due to its tissue specificity and incorporation into the ProstVac platform [9]. Prostatic acid phosphatase (PAP) served as the target for Sipuleucel-T, the first FDA-approved therapeutic cancer vaccine [10]. Cancer–testis antigens such as NY-ESO-1 are highly immunogenic and capable of inducing strong cellular and humoral responses [11].

MUC1 is another relevant shared antigen expressed across multiple GU malignancies, including renal cell, urothelial, and prostate cancers, where its expression correlates with advanced disease and poor outcomes [12,13]. Early vaccine strategies focused on tumor-associated antigens (TAAs), but these are limited by immune tolerance. Tumor-specific antigens (TSAs) or neoantigens, generated by somatic mutations, are more immunogenic and tumor-restricted, making them attractive vaccine targets [14].

Tumor mutational burden (TMB) influences neoantigen availability and varies across GU cancers. Prostate cancer generally has low TMB, urothelial carcinoma has high TMB, and renal cell carcinoma demonstrates intermediate levels [14]. Effective vaccine targets should generate strong peptide–MHC binding, activate CD8^+^ T cells, and ideally include multiple CD4^+^ and CD8^+^ epitopes to promote coordinated immune responses and antigen spreading [14].

### 2.2. Tumor Heterogeneity and Antigen Loss Variants

Intratumoral heterogeneity is a major barrier to vaccine efficacy in GU cancers. Prostate and renal cell carcinomas display significant genetic and phenotypic diversity, contributing to treatment resistance and variable responses to immunotherapy [15].

Many tumors contain subclonal neoantigens which arise from later branch mutations rather than truncal neoantigens, which come from an early trunk mutation that occurred before major tumor branching, allowing antigen-negative clones to escape immune pressure [16]. Vaccines targeting early, clonally expressed truncal neoantigens are therefore more likely to achieve durable responses. However, dynamic antigenic evolution and variability in MHC presentation can limit vaccine effectiveness over time [17]. These challenges support adaptive, multi-epitope vaccine strategies [16].

Earlier vaccines targeting self-antigens such as MART-1, gp100, and AFP produced immune responses but limited clinical benefit, emphasizing the need for broader polyclonal immunity. Some self-antigens may also suppress immune function; for example, AFP impairs dendritic cell activity [18].

### 2.3. Limitations in Immune Trafficking and Priming

Effective vaccine-induced immunity requires efficient immune cell trafficking and priming, processes that are frequently constrained in GU malignancies by tumor-intrinsic factors and the tumor microenvironment (TME). In prostate cancer, the TME comprises cancer-associated fibroblasts (CAFs), immune cells, extracellular matrix (ECM) components, and soluble mediators. This stromal architecture imposes both physical and biochemical barriers to immune cell infiltration and antigen presentation, thereby promoting immune evasion [19].

CAFs secrete immunomodulatory factors including TGF-β, IL-6, hepatocyte growth factor, and VEGF, which promote tumor growth, abnormal vasculature, and stromal remodeling while reinforcing immune exclusion. Excessive ECM and collagen deposition increases stromal rigidity and physically restricts lymphocyte trafficking into tumor nests [20]. Although immune cells are present within the prostate TME, chronic inflammatory signaling and stromal-derived mediators often render them dysfunctional [19].

Hypoxia represents a major structural and functional barrier within the bladder cancer TME. Rapid tumor proliferation outpaces vascular development, resulting in inefficient vasculature and regions of low oxygen tension. Hypoxic conditions promote tumor invasion, metabolic adaptation, and immune dysregulation. Hypoxia-inducible factors HIF-1α and HIF-2α directly modulate immune-related gene expression, influencing interferon signaling, humoral immunity, and Toll-like receptor pathways [21,22].

In renal cell carcinoma, tumor-infiltrating dendritic cells undergo metabolic reprogramming characterized by lipid accumulation, which impairs antigen processing and presentation. This dysfunction compromises effective T-cell priming despite immune cell infiltration [23].

### 2.4. Immunosuppressive Tumor Microenvironment

**Figure 1:**
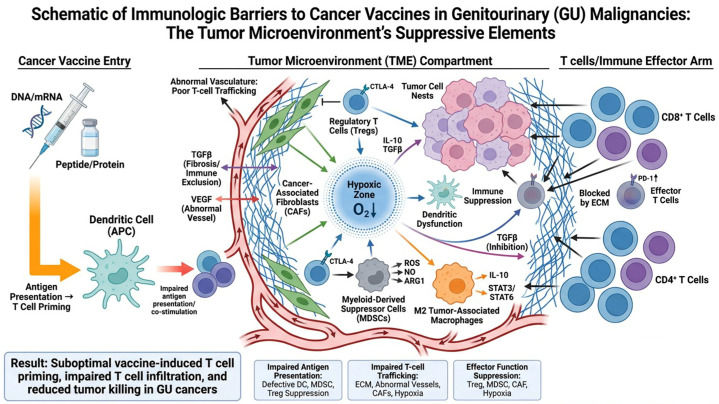
Immunologic barriers in genitourinary cancers relevant to vaccine development.

GU malignancies are characterized by a tumor microenvironment enriched in immunosuppressive cell populations, including regulatory T cells (Tregs), myeloid-derived suppressor cells (MDSCs), and M2-polarized tumor-associated macrophages (TAMs). These populations interact through cytokine signaling, metabolic suppression, and chemokine gradients to establish a self-reinforcing immunosuppressive milieu (Figure 1).

Tregs suppress effector T-cell activation through expression of immune checkpoint molecules such as CTLA-4 and secretion of IL-10 and TGF-β [24]. MDSCs, which increase with disease progression in prostate cancer, inhibit T-cell function through reactive oxygen species, nitric oxide production, metabolic depletion, and cytokine signaling [24]. Concurrently, TAMs adopt an M2 phenotype that promotes tumor progression and immune suppression via STAT3/STAT6-dependent pathways and immunosuppressive cytokines [24].

Immunosuppressive cytokine networks dominated by IL-10, TGF-β, and VEGF further limit antigen presentation, immune activation, and lymphocyte infiltration [25]. TGF-β also contributes to stromal fibrosis and immune exclusion, while VEGF-driven abnormal angiogenesis sustains hypoxia and impairs dendritic cell maturation [25]. In urothelial carcinoma, elevated TGF-β signaling is associated with cytotoxic T-cell exclusion and resistance to immune checkpoint blockade [20].

As a functional consequence, antigen presentation within the TME is impaired through defective dendritic cell maturation, reduced costimulatory signaling, and compromised cross-presentation, resulting in suboptimal cytotoxic T-cell responses [26]. Tumor cells may further evade immune recognition through MHC-I downregulation, alterations in antigen-processing machinery, and loss of neoantigen expression [27].

Structural barriers including dense ECM, collagen deposition, and endothelial dysfunction restrict T-cell infiltration and promote an immune-excluded phenotype [20,26]. Collectively, these features limit the efficacy of cancer vaccines by impairing antigen presentation, T-cell priming, and intratumoral trafficking. Clinically, advanced disease stage and older age are associated with more pronounced immunosuppressive mechanisms and diminished vaccine responsiveness [16].

### 2.5. Safety Considerations: Off-Target Immune Activation

Therapeutic cancer vaccines carry inherent risks of off-tumor immune activation due to peptide sequence homology with normal human proteins. Many TAAs are not uniquely tumor-specific and share identical epitopes with proteins expressed in healthy tissues. Consequently, vaccine-induced T-cell responses may cross-react with non-malignant cells, leading to immune-mediated tissue damage [28].

A clinically relevant example is provided by BNT112, an mRNA vaccine targeting prostate-restricted antigens. One of its targets, KLK2, shares immunogenic epitopes with KLK1, which is highly expressed in normal pancreatic tissue. Given the higher expression of KLK1 in the pancreas relative to KLK2 in prostate tumors, vaccine-induced T-cell responses may preferentially target pancreatic tissue, highlighting the importance of epitope specificity and safety evaluation in vaccine design [28].

## 3. Therapeutic Vaccine Platforms in GU Cancers

**Figure 2:**
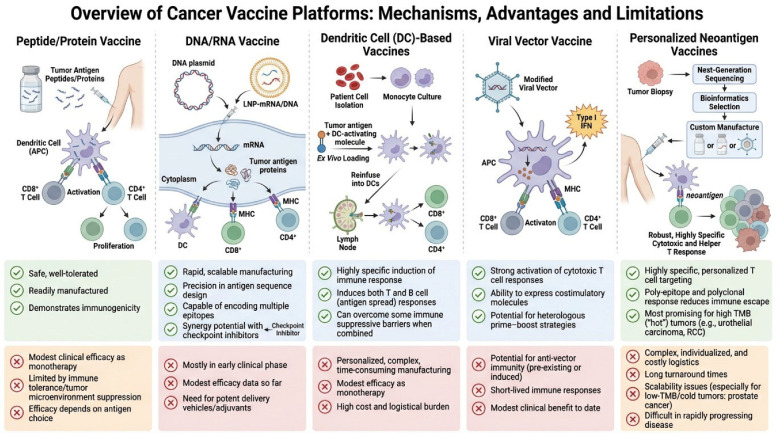
Vaccine platforms in GU cancers.

Figure 2 illustrates cancer vaccine platforms and mechanisms in GU malignancies.

### 3.1. Peptide and Protein Vaccines

In prostate cancer, peptide-based vaccines targeting PSA have been shown to induce cytotoxic T-cell responses against prostate cancer-associated antigens [29], but phase II trials did not demonstrate clinical benefit [30]. NY-ESO-1 is a tumor-associated antigen within the cancer–testis antigen (CTA) family and is expressed in multiple solid tumors, including bladder cancers [31] and prostate cancers [32]. In prostate cancer, vaccination with recombinant NY-ESO-1 induced antigen-specific immune responses in phase I studies and primed T-cell responses even in NY-ESO-1-antigen-negative patients [33], with evidence of prolonged PSA doubling time [34]. In bladder cancer, recombinant NY-ESO-1 protein vaccines administered with BCG and GM-CSF similarly induced antibody and CD4^+^ T-cell responses with favorable safety profiles, though CD8^+^ cytotoxic responses were less common [35].

Although these studies support the immunogenic potential of NY-ESO-1-targeted vaccines, clinical efficacy as monotherapy has remained limited, supporting the need for combination strategies with immune checkpoint inhibitors or other immunomodulators to strengthen antitumor activity [36,37]. The strong immunogenicity of NY-ESO-1, its expression in multiple genitourinary tumors, and its ability to elicit both humoral and cellular responses [32] make it an attractive candidate for combination immunotherapy. Nonetheless, immune tolerance and immunosuppression within the TME may limit clinical impact [34,35]. Overall, PSA- and NY-ESO-1-directed vaccines have demonstrated feasibility, safety, and immunogenicity and warrant continued development within modern immunotherapy paradigms for GU cancers.

### 3.2. DNA/RNA-Based Vaccines

mRNA-based cancer vaccines have been investigated extensively since a 1996 study showed that mice vaccinated with dendritic cells pulsed with RNA from ovalbumin (OVA)-expressing tumor cells were protected against challenge with OVA-expressing tumors [38]. The COVID-19 pandemic accelerated interest in mRNA platforms, and mRNA vaccines have emerged as a potentially useful approach in solid tumors by using synthetic mRNA encoding tumor-associated antigens to induce cellular and humoral immunity [39].

A total of 15 clinical trials are currently investigating mRNA-based cancer vaccines and may enroll patients with GU cancers (Table 1). Two trials specifically target KRAS-mutant tumors, while 13 enroll patients with advanced solid tumors irrespective of molecular or viral characteristics. Among these, seven evaluate mRNA vaccines in combination with another investigational therapy (most commonly PD-1 inhibitors), while eight assess monotherapy. Most are phase 1 or early phase 2 studies focused on safety, tolerability, and immunogenicity, with limited efficacy data currently available.

These early-phase trials include mRNA vaccines encoding tumor-associated antigens such as PSA, PSMA, and individualized neoantigens in prostate cancer, renal cell carcinoma, and bladder cancer. mRNA platforms enable rapid manufacturing, precise antigen selection, and potential synergy with immune checkpoint inhibitors.

Plasmid DNA vaccines encode tumor antigens in bacterial DNA constructs that are taken up by host cells and expressed to generate antigen-specific cellular and humoral immune responses [40]. In prostate cancer, a phase I trial of a plasmid encoding PSA demonstrated feasibility and safety and induced PSA-specific T-cell responses and IgG antibodies in hormone-refractory patients when administered with GM-CSF and IL-2 [41]. Similarly, plasmid DNA vaccines encoding PAP have elicited PAP-specific Th1-biased CD4^+^ and CD8^+^ responses in preclinical models [42]. Phase II studies of the PAP-encoding pTVG-HP DNA vaccine showed immunogenicity and safety in PSA-recurrent or castration-sensitive prostate cancer [43,44,45]. Ongoing studies are evaluating pTVG-HP in combination with immune checkpoint inhibitors such as nivolumab to strengthen antitumor immune responses [42,43,44,45].

### 3.3. Dendritic Cell-Based Vaccines

Sipuleucel-T is an autologous cellular immunotherapy for metastatic castration-resistant prostate cancer (mCRPC) that appears to act primarily through immune modulation rather than direct cytotoxicity. Ex vivo activation of antigen-presenting cells (APCs) with a PAP–GM-CSF fusion protein induces antigen-specific T-cell and B-cell responses upon reinfusion, and APC activation correlates with overall survival [7,46]. Treatment also promotes antigen spread, reflected by IgG responses to non-target tumor antigens, which are associated with improved outcomes [47]. In addition, systemic T-cell activation can recruit lymphocytes into the tumor microenvironment [48]. Biomarker analyses suggest that lower baseline PSA predicts greater benefit [49], while high-dimensional profiling of the infused product highlights lymphoid predominance and identifies effector subsets that may be enhanced by IL-15 co-stimulation [50]. These findings suggest that durable responses depend on effective APC priming, systemic T-cell activation, and antigen spread, offering principles for next-generation cellular and combination immunotherapies [51]. Clinically, sipuleucel-T improves overall survival with a favorable safety profile in randomized trials [46].

Dendritic cell (DC) vaccines have also been explored in renal cell carcinoma (RCC). Early-phase trials suggest DC vaccination is generally safe, can induce tumor-specific T-cell responses, and may yield occasional clinical responses, including stable disease and objective regressions in metastatic RCC [52,53]. Combining DC vaccines with targeted therapies such as sunitinib has been feasible and well tolerated and has been associated with reductions in immunosuppressive MDSCs and Tregs and detectable tumor-reactive T-cell responses [54,55]. Approaches integrating genetically modified DCs with cytokine-induced killer (CIK) cells in advanced RCC have demonstrated encouraging objective and disease control rates, suggesting improved antitumor activity compared with historical outcomes [55]. DC vaccination strategies combined with CIK cells or other systemic agents may improve disease control and survival metrics in metastatic and adjuvant contexts [56,57]. Despite progress, overall efficacy remains modest, emphasizing the need to optimize antigen specificity and integrate DC vaccines with rational immune-modulating combinations.

### 3.4. Viral Vector Vaccines

Viral vector vaccines are designed to deliver tumor-associated antigens in a manner that strongly activates T-cell responses and have emerged as a promising approach in GU cancers [58,59]. PROSTVAC, a poxviral vector targeting PSA and engineered with TRICOM costimulatory molecules, represents a leading example [60,61]. Early-phase studies suggested PROSTVAC could induce antigen-specific T-cell responses and potentially improve survival in mCRPC [62,63]. However, the subsequent phase III trial did not meet its primary endpoint. Potential contributors included short-lived immune responses, an immunosuppressive TME, preexisting anti-vector immunity, and suboptimal patient selection [60,62,64].

Next-generation viral vector vaccines aim to overcome these limitations through heterologous prime–boost strategies, incorporation of additional immune-stimulatory genes, improved vector engineering to reduce neutralizing immunity, neoantigen targeting, and rational combinations with checkpoint inhibitors or other immunomodulators [59,61]. These refinements seek to enhance both the magnitude and the durability of antitumor immunity and translate early immunogenicity into consistent clinical benefit [61,63].

### 3.5. Personalized Neoantigen Vaccines

Advances in sequencing and computational prediction have increased the feasibility of personalized neoantigen vaccines in RCC and urothelial carcinoma [65]. These approaches use tumor and normal tissue sequencing to identify somatic mutations and generate individualized vaccine constructs targeting predicted MHC-binding neoepitopes [65,66].

Early-phase studies in RCC demonstrated strong CD4^+^ and CD8^+^ T-cell responses against multiple neoantigens, even in heavily pretreated patients [67,68]. In bladder cancer, personalized vaccines combined with PD-1 blockade showed safety, feasibility, and clear neoantigen-specific immune activation [66,69]. Although challenges remain in sequencing, neoantigen prioritization, and manufacturing timelines, personalized vaccines represent a promising direction for precision immunotherapy in GU cancers.

## 4. Biological and Clinical Rationale for Vaccine Use in Specific GU Tumors

### 4.1. Prostate Cancer

Prostate cancer has a relatively low tumor mutational burden (TMB), contributing to an immunologically “cold” tumor microenvironment (TME) [70,71]. However, prostate tumors express shared antigens such as PAP and PSMA that are suitable targets for vaccine-based immunotherapy [72,73]. In metastatic castration-resistant prostate cancer (mCRPC), Sipuleucel-T improves survival by activating autologous APCs and stimulating CD4^+^ and CD8^+^ T-cell responses against PAP-expressing tumor cells [74,75].

The previously mentioned PROSTVAC induced PSA-specific T-cell responses in 57% of patients at 28 days post-vaccination, but active immunization ended at ~6 months while patients survived a median of 34.3 months—leaving years without antigenic stimulation. Long-term follow-up showed only 1 of 12 patients retained a detectable PSA-specific immune response at 66 months, consistent with rapid T-cell contraction. This suggests that the immune response was short-lived, contributing to the failure to improve clinical endpoints. The “cold” TME is harbored by Tregs, MDSCs, and SPP1hi tumor-associated macrophages that actively suppress CD8^+^ T-cell function, TGF-β, and checkpoint molecule expression. Even in localized prostate cancer—a far less hostile environment—neoadjuvant PROSTVAC produced only modest, statistically marginal increases in T-cell infiltration, suggesting the vaccine could not meaningfully remodel the TME of advanced disease. It should also be noted that the patient population was “unselected” with regards to biomarkers associated with immune responsiveness (e.g., microsatellite instability, tumor mutational burden), which actually only represents about 2–5% of mCRPCs.

The most promising path forward involves combination strategies with immune checkpoint inhibitors to simultaneously sustain vaccine-induced immunity and counteract the immunosuppressive TME. PROSTVAC combined with nivolumab increased CD4^+^ and CD8^+^ T-cell densities by more than two-fold in 91% and 83% of patients, respectively, with two mCRPC patients achieving prolonged complete radiographic responses—far exceeding monotherapy results. Concurrent (not sequential) PD-1 blockade with a DNA vaccine similarly demonstrated superior antitumor activity (62% vs. 8% with PSA declines; *p* = 0.01). Adenosine A2A receptor inhibition (ciforadenant) combined with PD-L1 blockade has shown clinical responses in mCRPC by reversing SPP1hi-TAM-mediated immunosuppression. Future trials should also incorporate biomarker-driven patient selection (MSI-H/dMMR, CDK12 biallelic loss, DDR deficiencies) to enrich for immunotherapy-responsive subsets, and consider earlier disease settings (e.g., metastatic hormone-sensitive disease) where tumor burden is lower and the TME is less immunosuppressive, as pilot combination trials in this space have shown a 2-year OS of 75%.

### 4.2. Renal Cell Carcinoma

Renal cell carcinoma (RCC) is considered immunogenic due to spontaneous T-cell infiltration and expression of antigens such as MUC1 and survivin [76]. However, vaccine responses are often limited by immunosuppressive factors including Tregs, MDSCs, TGF-β, and IL-10 [77,78].

Dendritic cell-based vaccines, alone or with cytokines such as IL-2, can stimulate tumor-specific CD4^+^ and CD8^+^ T-cell responses and promote Th1-mediated immunity [78,79]. Preclinical studies also suggest synergy between vaccines and checkpoint inhibitors, with combined approaches enhancing tumor control and T-cell activation [80]. Personalized neoantigen vaccines may further help overcome tumor heterogeneity and immune suppression [77,79].

### 4.3. Urothelial Carcinoma

Bladder cancer typically exhibits high TMB and abundant neoantigens, making it an attractive target for therapeutic vaccines in both BCG-relapsed non-muscle-invasive bladder cancer and advanced disease [81,82]. Neoantigen-directed vaccines can induce tumor-specific CD4^+^ and CD8^+^ immune responses [83], supporting development of peptide, dendritic cell, and personalized vaccine strategies [84,85].

Early-phase studies of vaccines such as S-288310, personalized peptide vaccination (PPV), and the PGV_001 neoantigen vaccine have demonstrated safety, immunogenicity, and preliminary clinical activity in patients with prior treatment failure [86,87,88]. These findings support combining vaccines with immune checkpoint inhibitors across different stages of urothelial carcinoma.

Nogapendekin alfa inbakicept is a first-in-class IL-15 receptor agonist (IL-15 superagonist fusion protein) that activates NK cells, CD8^+^ T cells, and memory T cells without expanding immunosuppressive Tregs, and is thought to synergize with BCG by enhancing BCG-induced immunity through a secondary immunostimulatory signal. It received FDA approval in April 2024 for intravesical use with BCG in BCG-unresponsive NMIBC, based on a single-arm trial by Chamie et al. reporting a CR rate of 71% with a median duration of 26.6 months. The safety profile was favorable, with most adverse events being grade 1–2 (86%); three grade 3 immune-related treatment-emergent adverse events occurred [89].

## 5. Overcoming Obstacles to Vaccine Success in GU Malignancies

### 5.1. Strategies to Address Antigen Heterogeneity

Antigen heterogeneity remains a major obstacle in GU vaccine therapy because variability in antigen expression across tumor cells can limit single-target vaccine efficacy and reduce overall immune control [90]. Multi-antigen (polyvalent) vaccines aim to broaden immune recognition and elicit polyclonal T-cell responses that cover diverse tumor subclones, improving protection against heterogeneous tumor populations [91,92]. Another strategy is the use of tumor-specific neoantigens derived from somatic mutations [93] to target each tumor’s unique antigenic composition. Combining these approaches with immune checkpoint inhibitors may further enhance T-cell infiltration and effector function in heterogeneous tumors [37,90,91].

### 5.2. Enhancing Immune Trafficking and Tumor Penetration

Radiotherapy increases tumor immunogenicity through antigen release and immunogenic cell death [94] and works synergistically with checkpoint blockade [95]. Targeting immunosuppressive stromal and myeloid populations can further improve immune infiltration and enhance vaccine responses after radiation [96].

In RCC, antiangiogenic therapy combined with checkpoint inhibitors is standard [97]. Low-dose antiangiogenic agents can reduce hypoxia and improve T-cell access [98], while vascular normalization (e.g., VEGF/VEGFR and ANG2/TIE2 pathways) may further support effective immune-mediated tumor killing [98].

### 5.3. Reducing Off-Tumor Immune Toxicity

Reducing off-tumor toxicity is critical in vaccine design due to shared antigen expression in GU cancers. Selecting tumor-restricted, highly immunogenic antigens lowers autoimmunity risk [90,91]. Localized delivery strategies, such as intratumoral or lymph node-targeted vaccines, can concentrate immune activation and limit systemic toxicity [95,99]. Personalized antigen selection further improves specificity and reduces off-target effects [92,100].

## 6. Combination Approaches: The Path Toward Effective Vaccine-Based Immunotherapy in GU Cancers

Personalized cancer vaccines target tumor-derived neoantigens to induce antigen-specific T-cell responses, enabling immune recognition of cancer, although optimal targets remain under investigation.

In metastatic disease with high tumor burden, neoantigen vaccines show limited efficacy, while they may be more effective in adjuvant settings by eliminating micrometastatic residual disease. In RCC, combination strategies are particularly important due to a highly immunosuppressive microenvironment, and optimal timing with immune checkpoint inhibitors is critical to maximizing T-cell priming and antitumor activity [66].

Cancer vaccines may help convert “cold” tumors into “hot” tumors, enhancing responsiveness to immunotherapy. Combining vaccines with checkpoint inhibitors can improve T-cell priming, expansion, and intratumoral activity, with generally limited added toxicity and potential synergy through increased PD-L1 expression and T-cell infiltration [101,102]. However, no FDA-approved vaccine–immunotherapy combination currently exists.

Radiotherapy can further enhance vaccine efficacy by inducing immunogenic tumor cell death and promoting antigen release. It also induces immunogenic modulation, including changes in antigen presentation and upregulation of death receptors (e.g., Fas/CD95), MHC class I, and adhesion molecules such as ICAM-1/CD54, increasing tumor susceptibility to T-cell-mediated killing [103,104].

**Figure 3:**
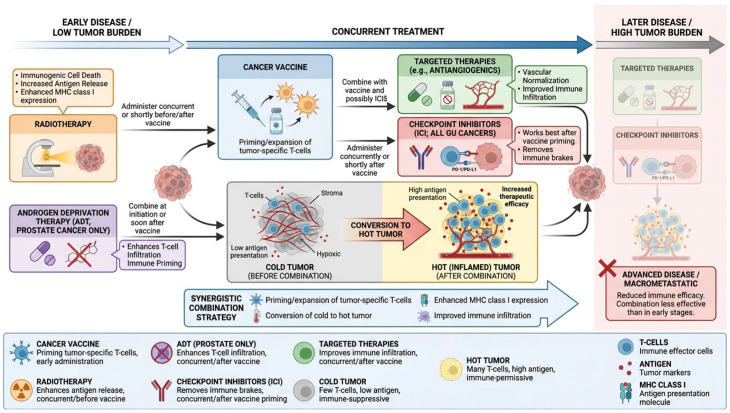
Combination strategies.

Figure 3 illustrates the different combination strategies used to enhance cancer vaccines efficacy in GU malignancies.

## 7. Future Directions

### 7.1. Precision Vaccine Development

Next-generation GU cancer vaccines increasingly rely on integrated genomic and immunopeptidomic approaches to improve neoantigen selection. Neoantigens derived from tumor-specific mutations can induce highly specific T-cell responses and are central to personalized vaccine design [105].

Standard pipelines use tumor–normal sequencing and RNA data to predict HLA-binding peptides, but in silico prediction alone is limited because many peptides are not naturally processed or presented [106,107]. This is particularly relevant in prostate cancer, where low TMB reduces available neoantigens [108].

Immunopeptidomics addresses this limitation by directly identifying MHC-bound peptides via mass spectrometry, improving antigen validity and reducing false positives [109]. In RCC, this approach has revealed previously unpredicted tumor-specific epitopes [110,111]. Integrated proteogenomic platforms such as NeoDisc and MHCquant2 further improve neoantigen prioritization, including detection of subclonal and noncanonical peptides [112,113].

Early RCC and urothelial carcinoma studies show that personalized neoantigen vaccines can induce durable CD4^+^ and CD8^+^ T-cell responses [66,114,115,116]. In prostate cancer, where TMB is low, immunopeptidomics may help identify alternative targets such as splice variants and noncanonical peptides [117,118,119].

Overall, integrating genomics, transcriptomics, and immunopeptidomics enables more accurate antigen selection and supports rational combination strategies with checkpoint inhibitors and other therapies.

### 7.2. Next-Generation Delivery Systems

Effective vaccine delivery is critical in GU cancers due to immune exclusion and stromal barriers [120]. Nanoparticle and liposomal systems improve antigen stability, lymphatic trafficking, and uptake by antigen-presenting cells, enhancing CD8^+^ T-cell priming [121,122].

Lipid nanoparticles (LNPs) enable co-delivery of antigens and adjuvants and can be tuned by size, charge, and composition to optimize lymph node targeting and immune activation [123,124,125]. Particle size influences biodistribution, while surface charge affects uptake and toxicity [126,127,128]. Ionizable lipids facilitate endosomal escape and antigen presentation via MHC I and II pathways, supporting both CD8^+^ and CD4^+^ responses [128,129,130].

Co-delivery of innate immune agonists (e.g., TLR or STING ligands) further enhances dendritic cell activation and antigen-specific T-cell responses [121,131,132].

Oncolytic viruses add another strategy by directly lysing tumor cells, releasing antigens, and creating an inflamed microenvironment that enhances immune priming [133,134]. Engineered viral vectors can also serve as in situ vaccine platforms and synergize with checkpoint blockade [135,136].

### 7.3. Rational Combination Regimens

Vaccine efficacy depends on timing, tumor burden, and immune status. Vaccines are most effective when immune priming capacity is intact, often earlier in disease or in lower tumor burden settings [115,116,137,138].

In prostate cancer, ADT enhances immune activation and supports vaccine responses, while late mCRPC is associated with immune exhaustion [9,139,140]. Sipuleucel-T demonstrates survival benefit despite minimal tumor shrinkage, consistent with immune-mediated effects [7].

Radiotherapy and radiopharmaceuticals enhance antigen release and immune visibility, supporting synergy with vaccines and checkpoint inhibitors [141,142,143].

In RCC and urothelial carcinoma, vaccines may convert “cold” tumors into inflamed phenotypes, increasing responsiveness to checkpoint blockade through T-cell expansion and PD-1/PD-L1 upregulation [144,145].

Biomarker selection is critical, as prior trials show benefit mainly in immune responders [146]. TMB is predictive in some tumors, but alternative markers such as immune infiltration and antigen presentation may be more relevant in prostate cancer and RCC [147,148,149,150,151,152,153,154].

### 7.4. Regulatory and Practical Considerations

Personalized vaccines face major manufacturing, regulatory, and economic challenges. Production requires sequencing, bioinformatic analysis, good manufacturing particle (GMP) synthesis, and individualized quality control [155,156]. Current timelines of 3–5 months limit use in rapidly progressive disease [157].

Scalability is constrained by patient-specific workflows and lack of standardized manufacturing processes [155,157]. Costs are high due to individualized production and lack of economies of scale, limiting reimbursement and access [158,159].

Solutions include semi-personalized platforms, shared neoantigen libraries, and modular manufacturing approaches that improve scalability and reduce cost while maintaining specificity [159,160].

## 8. Conclusions

Cancer vaccines are a new therapeutic modality that is promising for many cancer types. They can improve outcomes especially for “Cold” tumors like prostate cancer. Cancer vaccines can be used in combination treatment, such as with immune checkpoint inhibitors, to enhance antitumor activity by administering antigens conjugated with costimulatory molecules that eventually trigger the immune response [161] and active stimulation of tumor-associated antigens (TAAs). The mechanism in theory is very promising; however, no improvement in survival has been found in most studies so far, with very few approvals. Possible mechanisms that could explain this include immune escape mechanisms, or tumor heterogeneity, in which different TAAs can be present in different tumor areas [162].

Given persistent unmet needs in GU malignancies, therapeutic cancer vaccines remain an important avenue for investigation. Additional clinical and preclinical studies are needed to identify predictive biomarkers, optimize combinations and sequencing, and improve outcomes for patients with genitourinary cancers.

## Figures and Tables

**Table 1:** Current Clinical Trials of mRNA Vaccines in GU Malignancies.

NCT ID	Phase	Target Antigen/Vaccine	Cancer Type (Indication)	Combination or Monotherapy
NCT07004244	Phase I	KRAS-mutant neoantigen mRNA	KRAS-mutant tumors	Monotherapy
NCT06195384	Phase I	Personalized neoantigen mRNA	Advanced solid tumors	Monotherapy
NCT07245901	Phase I/II	Tumor-specific circular RNA antigen mRNA	Advanced solid tumors	Combination (with anti-PD-1)
NCT07040943	Phase I	IL-22BP mRNA vaccine	Refractory solid tumors	Monotherapy
NCT07182435	Phase I	Personalized neoantigen mRNA (RH125)	Advanced/metastatic tumors	Monotherapy
NCT07363369	Exploratory	FAP mRNA vaccine	Advanced solid tumors	Combination (with ICI)
NCT05949775	Phase I	Personalized neoantigen mRNA	Advanced solid tumors	Combination (with sintilimab)
NCT07100210	Phase I	IL-22BP mRNA	Advanced solid tumors	Monotherapy
NCT06577532	Phase I	KRAS neoantigen mRNA (ABO2102)	KRAS-mutant tumors	Combination (with toripalimab)
NCT06497010	Phase I	Personalized neoantigen mRNA	Advanced solid tumors	Monotherapy
NCT05916248	Phase I	Neoantigen mRNA vaccine	Advanced solid tumors	Monotherapy/possible ICI
NCT07348042	Phase I	RGL-270 personalized neoantigen mRNA	High-risk post-surgery solid tumors	Combination (with adebelimab)
NCT05579275	Phase I	JCXH-212 neoantigen mRNA	Advanced solid tumors	Combination (with toripalimab)
NCT07028047	Phase I	WCg-043 mRNA vaccine	Advanced solid tumors	Monotherapy
NCT07307053	Phase I/II	mRNA tumor antigen platform	Rare tumors	Platform/varied

## Data Availability

No new data were created or analyzed in this study.

## Abbreviations

GU	Genitourinary
TME	Tumor Microenvironment
TMB	Tumor Mutational Burden
PSA	Prostate-Specific Antigen
PAP	Prostatic Acid Phosphatase
MDSCs	Myeloid-Derived Suppressor Cells
Tregs	Regulatory T Cells
RCC	Renal Cell Carcinoma
NMIBC	Non-Muscle-Invasive Bladder Cancer
ICIs	Immune Checkpoint Inhibitors

## References

[1] DeMaria P.J., Bilusic M. (2019). Cancer Vaccines. Hematol. Oncol. Clin. N. Am..

[2] Lamm D.L., Thor D.E., Harris S.C., Reyna J.A., Stogdill V.D., Radwin H.M. (1980). Bacillus Calmette-Guerin immunotherapy of superficial bladder cancer. J. Urol..

[3] Fyfe G., Fisher R.I., Rosenberg S.A., Sznol M., Parkinson D.R., Louie A.C. (1995). Results of treatment of 255 patients with metastatic renal cell carcinoma who received high-dose recombinant interleukin-2 therapy. J. Clin. Oncol..

[4] Rini B.I., Halabi S., Rosenberg J.E., Stadler W.M., Vaena D.A., Archer L., Atkins J.N., Picus J., Czaykowski P., Dutcher J. (2010). Phase III trial of bevacizumab plus interferon alfa versus interferon alfa monotherapy in patients with metastatic renal cell carcinoma: Final results of CALGB 90206. J. Clin. Oncol..

[5] Motzer R.J., Escudier B., McDermott D.F., George S., Hammers H.J., Srinivas S., Tykodi S.S., Sosman J.A., Procopio G., Plimack E.R. (2015). Nivolumab versus Everolimus in Advanced Renal-Cell Carcinoma. N. Engl. J. Med..

[6] Powles T., Eder J.P., Fine G.D., Braiteh F.S., Loriot Y., Cruz C., Bellmunt J., Burris H.A., Petrylak D.P., Teng S.-L. (2014). MPDL3280A (anti-PD-L1) treatment leads to clinical activity in metastatic bladder cancer. Nature.

[7] Kantoff P.W., Higano C.S., Shore N.D., Berger E.R., Small E.J., Penson D.F., Redfern C.H., Ferrari A.C., Dreicer R., Sims R.B. (2010). Sipuleucel-T immunotherapy for castration-resistant prostate cancer. N. Engl. J. Med..

[8] Mehta K., Patel K., Parikh R.A. (2017). Immunotherapy in genitourinary malignancies. J. Hematol. Oncol..

[9] Drake C.G. (2010). Prostate cancer as a model for tumour immunotherapy. Nat. Rev. Immunol..

[10] Cheever M.A., Higano C.S. (2011). PROVENGE (Sipuleucel-T) in Prostate Cancer: The First FDA-Approved Therapeutic Cancer Vaccine. Clin. Cancer Res..

[11] Alsalloum A., Shevchenko J.A., Sennikov S. (2024). NY-ESO-1 antigen: A promising frontier in cancer immunotherapy. Clin. Transl. Med..

[12] Grewal U.S., Kurzrock R. (2025). Mucin-1: A promising pan-cancer therapeutic target. npj Precis. Oncol..

[13] Mao W., Zhang H., Wang K., Geng J., Wu J. (2024). Research progress of MUC1 in genitourinary cancers. Cell. Mol. Biol. Lett..

[14] Zhao Y., Baldin A.V., Isayev O., Werner J., Zamyatnin A.A., Bazhin A.V. (2021). Cancer Vaccines: Antigen Selection Strategy. Vaccines.

[15] Zhu S., Chen J., Zeng H. (2023). Our Current Understanding of the Heterogeneity in Prostate Cancer and Renal Cell Carcinoma. J. Clin. Med..

[16] Pounraj S., Chen S., Ma L., Mazzieri R., Dolcetti R., Rehm B.H.A. (2024). Targeting Tumor Heterogeneity with Neoantigen-Based Cancer Vaccines. Cancer Res..

[17] Aljabali A.A.A., Hamzat Y., Alqudah A., Alzoubi L. (2025). Neoantigen vaccines: Advancing personalized cancer immunotherapy. Explor. Immunol..

[18] Butterfield L.H. (2016). Lessons learned from cancer vaccine trials and target antigen choice. Cancer Immunol. Immunother..

[19] Bahmad H.F., Jalloul M., Azar J., Moubarak M.M., Samad T.A., Mukherji D., Al-Sayegh M., Abou-Kheir W. (2021). Tumor Microenvironment in Prostate Cancer: Toward Identification of Novel Molecular Biomarkers for Diagnosis, Prognosis, and Therapy Development. Front. Genet..

[20] Chung S.W., Xie Y., Suk J.S. (2021). Overcoming physical stromal barriers to cancer immunotherapy. Drug Deliv. Transl. Res..

[21] Peixoto A., Fernandes E., Gaiteiro C., Lima L., Azevedo R., Soares J., Cotton S., Parreira B., Neves M., Amaro T. (2016). Hypoxia enhances the malignant nature of bladder cancer cells and concomitantly antagonizes protein O-glycosylation extension. Oncotarget.

[22] Smith V., Lee D., Reardon M., Shabbir R., Sahoo S., Hoskin P., Choudhury A., Illidge T., West C.M.L. (2023). Hypoxia Is Associated with Increased Immune Infiltrates and Both Anti-Tumour and Immune Suppressive Signalling in Muscle-Invasive Bladder Cancer. Int. J. Mol. Sci..

[23] Herber D.L., Cao W., Nefedova Y., Novitskiy S.V., Nagaraj S., Tyurin V.A., Corzo A., Cho H.-I., Celis E., Lennox B. (2010). Lipid accumulation and dendritic cell dysfunction in cancer. Nat. Med..

[24] Huang J., Ojo A., Tsao S., Horowitz A., Kyprianou N., Tsao C.-K. (2025). Overcoming Immune Evasion in the Prostate Tumor Microenvironment: Novel Targeted Strategies to Improve Treatment Outcomes. Cancers.

[25] Yi M., Li T., Niu M., Zhang H., Wu Y., Wu K., Dai Z. (2024). Targeting cytokine and chemokine signaling pathways for cancer therapy. Signal Transduct. Target. Ther..

[26] Bandola-Simon J., Roche P.A. (2019). Dysfunction of antigen processing and presentation by dendritic cells in cancer. Mol. Immunol..

[27] Ferrone S., Marincola F.M. (1995). Loss of HLA class I antigens by melanoma cells: Molecular mechanisms, functional significance and clinical relevance. Immunol. Today.

[28] Kellermann G., Mograbi B., Hofman P., Brest P. (2025). Shared epitopes create safety and efficacy concerns in several cancer vaccines. J. Immunother. Cancer.

[29] Xue B.H., Zhang Y., Sosman J.A., Peace D.J. (1997). Induction of human cytotoxic T lymphocytes specific for prostate-specific antigen. Prostate.

[30] Kouiavskaia D.V., Berard C.A., Datena E., Hussain A., Dawson N., Klyushnenkova E.N., Alexander R.B. (2009). Vaccination with agonist peptide PSA: 154-163 (155L) derived from prostate specific antigen induced CD8 T-cell response to the native peptide PSA: 154-163 but failed to induce the reactivity against tumor targets expressing PSA: A phase 2 study in patients with recurrent prostate cancer. J. Immunother..

[31] Sharma P., Gnjatic S., Jungbluth A.A., Williamson B., Herr H., Stockert E., Dalbagni G., Donat S.M., Reuter V.E., Santiago D. (2003). Frequency of NY-ESO-1 and LAGE-1 expression in bladder cancer and evidence of a new NY-ESO-1 T-cell epitope in a patient with bladder cancer. Cancer Immun..

[32] Fossa A., Berner A., Fossa S.D., Hernes E., Gaudernack G., Smeland E.B. (2004). NY-ESO-1 protein expression and humoral immune responses in prostate cancer. Prostate.

[33] Karbach J., Neumann A., Atmaca A., Wahle C., Brand K., von Boehmer L., Knuth A., Bender A., Ritter G., Old L.J. (2011). Efficient in vivo priming by vaccination with recombinant NY-ESO-1 protein and CpG in antigen naive prostate cancer patients. Clin. Cancer Res..

[34] Sonpavde G., Wang M., Peterson L.E., Wang H.Y., Joe T., Mims M.P., Kadmon D., Ittmann M.M., Wheeler T.M., Gee A.P. (2014). HLA-restricted NY-ESO-1 peptide immunotherapy for metastatic castration resistant prostate cancer. Investig. New Drugs.

[35] Sharma P., Bajorin D.F., Jungbluth A.A., Herr H., Old L.J., Gnjatic S. (2008). Immune responses detected in urothelial carcinoma patients after vaccination with NY-ESO-1 protein plus BCG and GM-CSF. J. Immunother..

[36] Sahin U., Oehm P., Derhovanessian E., Jabulowsky R.A., Vormehr M., Gold M., Maurus D., Schwarck-Kokarakis D., Kuhn A.N., Omokoko T. (2020). An RNA vaccine drives immunity in checkpoint-inhibitor-treated melanoma. Nature.

[37] Xue W., Metheringham R.L., Brentville V.A., Gunn B., Symonds P., Yagita H., Ramage J.M., Durrant L.G. (2016). SCIB2, an antibody DNA vaccine encoding NY-ESO-1 epitopes, induces potent antitumor immunity which is further enhanced by checkpoint blockade. Oncoimmunology.

[38] Boczkowski D., Nair S.K., Snyder D., Gilboa E. (1996). Dendritic cells pulsed with RNA are potent antigen-presenting cells in vitro and in vivo. J. Exp. Med..

[39] Lorentzen C.L., Haanen J.B., Met O., Svane I.M. (2022). Clinical advances and ongoing trials on mRNA vaccines for cancer treatment. Lancet Oncol..

[40] Liu M.A. (2003). DNA vaccines: A review. J. Intern. Med..

[41] Pavlenko M., Roos A.-K., Lundqvist A., Palmborg A., Miller A.M., Ozenci V., Bergman B., Egevad L., Hellström M., Kiessling R. (2004). A phase I trial of DNA vaccination with a plasmid expressing prostate-specific antigen in patients with hormone-refractory prostate cancer. Br. J. Cancer.

[42] Johnson L.E., Frye T.P., Chinnasamy N., Chinnasamy D., McNeel D.G. (2007). Plasmid DNA vaccine encoding prostatic acid phosphatase is effective in eliciting autologous antigen-specific CD8^+^ T cells. Cancer Immunol. Immunother..

[43] McNeel D.G., Eickhoff J.C., Johnson L.E., Roth A.R., Perk T.G., Fong L., Antonarakis E.S., Wargowski E., Jeraj R., Liu G. (2019). Phase II Trial of a DNA Vaccine Encoding Prostatic Acid Phosphatase (pTVG-HP [MVI-816]) in Patients With Progressive, Nonmetastatic, Castration-Sensitive Prostate Cancer. J. Clin. Oncol..

[44] Tonelli T.P., Eickhoff J.C., Johnson L.E., Liu G., McNeel D.G. (2024). Long-term follow up of patients treated with a DNA vaccine (pTVG-hp) for PSA-recurrent prostate cancer. Hum. Vaccines Immunother..

[45] McNeel D.G., Emamekhoo H., Eickhoff J.C., Kyriakopoulos C.E., Wargowski E., Tonelli T.P., Johnson L.E., Liu G. (2023). Phase 2 trial of a DNA vaccine (pTVG-HP) and nivolumab in patients with castration-sensitive non-metastatic (M0) prostate cancer. J. Immunother. Cancer.

[46] Sheikh N.A., Petrylak D., Kantoff P.W., dela Rosa C., Stewart F.P., Kuan L.-Y., Whitmore J.B., Trager J.B., Poehlein C.H., Frohlich M.W. (2013). Sipuleucel-T immune parameters correlate with survival: An analysis of the randomized phase 3 clinical trials in men with castration-resistant prostate cancer. Cancer Immunol. Immunother..

[47] GuhaThakurta D., Sheikh N.A., Fan L.Q., Kandadi H., Meagher T.C., Hall S.J., Kantoff P.W., Higano C.S., Small E.J., Gardner T.A. (2015). Humoral Immune Response against Nontargeted Tumor Antigens after Treatment with Sipuleucel-T and Its Association with Improved Clinical Outcome. Clin. Cancer Res..

[48] Fong L., Carroll P., Weinberg V., Chan S., Lewis J., Corman J., Amling C.L., Stephenson R.A., Simko J., Sheikh N.A. (2014). Activated lymphocyte recruitment into the tumor microenvironment following preoperative sipuleucel-T for localized prostate cancer. J. Natl. Cancer Inst..

[49] Schellhammer P.F., Chodak G., Whitmore J.B., Sims R., Frohlich M.W., Kantoff P.W. (2013). Lower baseline prostate-specific antigen is associated with a greater overall survival benefit from sipuleucel-T in the Immunotherapy for Prostate Adenocarcinoma Treatment (IMPACT) trial. Urology.

[50] Saeed M.A., Peng B., Kim K., Rawat K., Kuehm L.M., Siegel Z.R., Borkowski A., Habib N., Van Tine B., Sheikh N. (2024). High-Dimensional Analyses Reveal IL15 Enhances Activation of Sipuleucel-T Lymphocyte Subsets and Reverses Immunoresistance. Cancer Immunol. Res..

[51] Higano C.S., Armstrong A.J., Sartor A.O., Vogelzang N.J., Kantoff P.W., McLeod D.G., Pieczonka C.M., Penson D.F., Shore N.D., Vacirca J. (2019). Real-world outcomes of sipuleucel-T treatment in PROCEED, a prospective registry of men with metastatic castration-resistant prostate cancer. Cancer.

[52] Berntsen A., Geertsen P.F., Svane I.M. (2006). Therapeutic dendritic cell vaccination of patients with renal cell carcinoma. Eur. Urol..

[53] Berntsen A., Trepiakas R., Wenandy L., Geertsen P.F., thor Straten P., Andersen M.H., Pedersen A.E., Claesson M.H., Lorentzen T., Johansen J. (2008). Therapeutic dendritic cell vaccination of patients with metastatic renal cell carcinoma: A clinical phase 1/2 trial. J. Immunother..

[54] Matsushita H., Enomoto Y., Kume H., Nakagawa T., Fukuhara H., Suzuki M., Fujimura T., Homma Y., Kakimi K. (2014). A pilot study of autologous tumor lysate-loaded dendritic cell vaccination combined with sunitinib for metastatic renal cell carcinoma. J. Immunother. Cancer.

[55] Wang D., Zhang B., Gao H., Ding G., Wu Q., Zhang J., Liao L., Chen H. (2014). Clinical research of genetically modified dendritic cells in combination with cytokine-induced killer cell treatment in advanced renal cancer. BMC Cancer.

[56] Zhang Y., Ellinger J., Ritter M., Schmidt-Wolf I.G.H. (2020). Clinical Studies Applying Cytokine-Induced Killer Cells for the Treatment of Renal Cell Carcinoma. Cancers.

[57] Liu L., Zhang W., Qi X., Li H., Yu J., Wei S., Hao X., Ren X. (2012). Randomized study of autologous cytokine-induced killer cell immunotherapy in metastatic renal carcinoma. Clin. Cancer Res..

[58] Larocca C., Schlom J. (2011). Viral vector-based therapeutic cancer vaccines. Cancer J..

[59] Seclì L., Leoni G., Ruzza V., Siani L., Cotugno G., Scarselli E., D’alise A.M. (2023). Personalized Cancer Vaccines Go Viral: Viral Vectors in the Era of Personalized Immunotherapy of Cancer. Int. J. Mol. Sci..

[60] Singh P., Pal S.K., Alex A., Agarwal N. (2015). Development of PROSTVAC immunotherapy in prostate cancer. Future Oncol..

[61] Sater H.A., Marté J.L., Donahue R.N., Walter-Rodriguez B., Heery C.R., Steinberg S.M., Cordes L.M., Chun G., Karzai F., Bilusic M. (2020). Neoadjuvant PROSTVAC prior to radical prostatectomy enhances T-cell infiltration into the tumor immune microenvironment in men with prostate cancer. J. Immunother. Cancer.

[62] Madan R.A., Arlen P.M., Mohebtash M., Hodge J.W., Gulley J.L. (2009). Prostvac-VF: A vector-based vaccine targeting PSA in prostate cancer. Expert Opin. Investig. Drugs.

[63] Gulley J.L., Borre M., Vogelzang N.J., Ng S., Agarwal N., Parker C.C., Pook D.W., Rathenborg P., Flaig T.W., Carles J. (2019). Phase III Trial of PROSTVAC in Asymptomatic or Minimally Symptomatic Metastatic Castration-Resistant Prostate Cancer. J. Clin. Oncol..

[64] Madan R.A., Bilusic M., Stein M.N., Donahue R.N., Arlen P.M., Karzai F., Plimack E., Wong Y.-N., Geynisman D.M., Zibelman M. (2023). Flutamide With or Without PROSTVAC in Non-metastatic Castration Resistant (M0) Prostate Cancer. Oncologist.

[65] Ott P.A., Hu-Lieskovan S., Chmielowski B., Govindan R., Naing A., Bhardwaj N., Margolin K., Awad M.M., Hellmann M.D., Lin J.J. (2020). A Phase Ib Trial of Personalized Neoantigen Therapy Plus Anti-PD-1 in Patients with Advanced Melanoma, Non-small Cell Lung Cancer, or Bladder Cancer. Cell.

[66] Braun D.A., Moranzoni G., Chea V., McGregor B.A., Blass E., Tu C.R., Vanasse A.P., Forman C., Forman J., Afeyan A.B. (2025). A neoantigen vaccine generates antitumour immunity in renal cell carcinoma. Nature.

[67] Zeng Y., Zhang W., Li Z., Zheng Y., Wang Y., Chen G., Qiu L., Ke K., Su X., Cai Z. (2020). Personalized neoantigen-based immunotherapy for advanced collecting duct carcinoma: Case report. J. Immunother. Cancer.

[68] Peshin S., Dharia A., Moka N., Skelton W.P.t. (2025). A Review of Immunotherapy in Renal Cell Carcinoma: Current Landscape and Future Directions. Cancers.

[69] Zaidi N. (2020). Can Personalized Neoantigens Raise the T Cell Bar?. Cell.

[70] Samija I., Frobe A. (2019). Challenges in Manipulating Immune System to Treat Prostate Cancer. Acta Clin. Croat..

[71] Runcie K.D., Dallos M.C. (2021). Prostate Cancer Immunotherapy-Finally in From the Cold?. Curr. Oncol. Rep..

[72] Patel D., McKay R., Parsons J.K. (2020). Immunotherapy for Localized Prostate Cancer: The Next Frontier?. Urol. Clin. N. Am..

[73] Xu P., Wasielewski L.J., Yang J.C., Cai D., Evans C.P., Murphy W.J., Liu C. (2022). The Immunotherapy and Immunosuppressive Signaling in Therapy-Resistant Prostate Cancer. Biomedicines.

[74] Burotto M., Singh N., Heery C.R., Gulley J.L., Madan R.A. (2014). Exploiting synergy: Immune-based combinations in the treatment of prostate cancer. Front. Oncol..

[75] Kalina J.L., Neilson D.S., Comber A.P., Rauw J.M., Alexander A.S., Vergidis J., Lum J.J. (2017). Immune Modulation by Androgen Deprivation and Radiation Therapy: Implications for Prostate Cancer Immunotherapy. Cancers.

[76] Meng L., Collier K.A., Wang P., Li Z., Monk P., Mortazavi A., Hu Z., Spakowicz D., Zheng L., Yang Y. (2023). Emerging Immunotherapy Approaches for Advanced Clear Cell Renal Cell Carcinoma. Cells.

[77] Oudard S., Rixe O., Beuselinck B., Linassier C., Banu E., Machiels J.-P., Baudard M., Ringeisen F., Velu T., Lefrere-Belda M.-A. (2011). A phase II study of the cancer vaccine TG4010 alone and in combination with cytokines in patients with metastatic renal clear-cell carcinoma: Clinical and immunological findings. Cancer Immunol. Immunother..

[78] Baek S., Kim C.-S., Kim S.-B., Kim Y.-M., Kwon S.-W., Kim Y., Kim H., Lee H. (2011). Combination therapy of renal cell carcinoma or breast cancer patients with dendritic cell vaccine and IL-2: Results from a phase I/II trial. J. Transl. Med..

[79] Palucka K., Banchereau J. (2012). Cancer immunotherapy via dendritic cells. Nat. Rev. Cancer.

[80] Ueki H., Kitagawa K., Kato M., Yanase S., Okamura Y., Bando Y., Hara T., Terakawa T., Furukawa J., Nakano Y. (2023). An oral cancer vaccine using Bifidobacterium vector augments combination of anti-PD-1 and anti-CTLA-4 antibodies in mouse renal cell carcinoma model. Sci. Rep..

[81] Burger M., Catto J.W.F., Dalbagni G., Grossman H.B., Herr H., Karakiewicz P., Kassouf W., Kiemeney L.A., La Vecchia C., Shariat S. (2013). Epidemiology and risk factors of urothelial bladder cancer. Eur. Urol..

[82] Lang F., Schrörs B., Löwer M., Türeci Ö., Sahin U. (2022). Identification of neoantigens for individualized therapeutic cancer vaccines. Nat. Rev. Drug Discov..

[83] Oh D.Y., Kwek S.S., Raju S.S., Li T., McCarthy E., Chow E., Aran D., Ilano A., Pai C.-C.S., Rancan C. (2020). Intratumoral CD4(+) T Cells Mediate Anti-tumor Cytotoxicity in Human Bladder Cancer. Cell.

[84] Zhang H., Hong H., Li D., Ma S., Di Y., Stoten A., Haig N., Di Gleria K., Yu Z., Xu X.-N. (2009). Comparing Pooled Peptides with Intact Protein for Accessing Cross-presentation Pathways for Protective CD8^+^ and CD4^+^ T Cells. J. Biol. Chem..

[85] Hu Z., Leet D.E., Allesøe R.L., Oliveira G., Li S., Luoma A.M., Liu J., Forman J., Huang T., Iorgulescu J.B. (2021). Personal neoantigen vaccines induce persistent memory T cell responses and epitope spreading in patients with melanoma. Nat. Med..

[86] Saxena M., Marron T., Kodysh J., Rubinsteyn A., Finnigan J., Blasquez A., Meseck M., O’DOnnell T., Delbeau D., Galsky M. (2023). Abstract CT270: Immunogenicity of PGV_001 neoantigen vaccine in a Phase-I clinical trial, across various types of cancers in adjuvant setting. Cancer Res..

[87] Saxena M., Anker J.F., Kodysh J., O’dOnnell T., Kaminska A.M., Meseck M., Hapanowicz O., Niglio S.A., Salazar A.M., Shah H.R. (2025). Atezolizumab plus personalized neoantigen vaccination in urothelial cancer: A phase 1 trial. Nat. Cancer.

[88] Liu J., Fu M., Wang M., Wan D., Wei Y., Wei X. (2022). Cancer vaccines as promising immuno-therapeutics: Platforms and current progress. J. Hematol. Oncol..

[89] Chamie K., Chang S.S., Kramolowsky E., Gonzalgo M.L., Agarwal P.K., Bassett J.C., Bjurlin M., Cher M.L., Clark W., Cowan B.E. (2023). IL-15 Superagonist NAI in BCG-Unresponsive Non-Muscle-Invasive Bladder Cancer. NEJM Evid..

[90] Ye Z., Qian Q., Jin H., Qian Q. (2018). Cancer vaccine: Learning lessons from immune checkpoint inhibitors. J. Cancer.

[91] Tagliamonte M., Petrizzo A., Tornesello M.L., Buonaguro F.M., Buonaguro L. (2014). Antigen-specific vaccines for cancer treatment. Hum. Vaccines Immunother..

[92] Sayour E.J., Mitchell D.A. (2017). Manipulation of Innate and Adaptive Immunity through Cancer Vaccines. J. Immunol. Res..

[93] Heemskerk B., Kvistborg P., Schumacher T.N. (2013). The cancer antigenome. EMBO J..

[94] Reits E.A., Hodge J.W., Herberts C.A., Groothuis T.A., Chakraborty M., Wansley E.K., Camphausen K., Luiten R.M., De Ru A.H., Neijssen J. (2006). Radiation modulates the peptide repertoire, enhances MHC class I expression, and induces successful antitumor immunotherapy. J. Exp. Med..

[95] Zhu S., Zhang T., Zheng L., Liu H., Song W., Liu D., Li Z., Pan C.-X. (2021). Combination strategies to maximize the benefits of cancer immunotherapy. J. Hematol. Oncol..

[96] Wu C.Y., Yang L.H., Yang H.Y., Knoff J., Peng S., Lin Y.H., Wang C., Alvarez R.D., Pai S.I., Roden R.B.S. (2014). Enhanced cancer radiotherapy through immunosuppressive stromal cell destruction in tumors. Clin. Cancer Res..

[97] Mantia C.M., McDermott D.F. (2019). Vascular endothelial growth factor and programmed death-1 pathway inhibitors in renal cell carcinoma. Cancer.

[98] Dianat-Moghadam H., Nedaeinia R., Keshavarz M., Azizi M., Kazemi M., Salehi R. (2023). Immunotherapies targeting tumor vasculature: Challenges and opportunities. Front. Immunol..

[99] Sridaran D., Bradshaw E., DeSelm C., Pachynski R., Mahajan K., Mahajan N.P. (2023). Prostate cancer immunotherapy: Improving clinical outcomes with a multi-pronged approach. Cell Rep. Med..

[100] Alizadeh A.A., Aranda V., Bardelli A., Blanpain C., Bock C., Borowski C., Caldas C., Califano A., Doherty M., Elsner M. (2015). Toward understanding and exploiting tumor heterogeneity. Nat. Med..

[101] Collins J.M., Redman J.M., Gulley J.L. (2018). Combining vaccines and immune checkpoint inhibitors to prime, expand, and facilitate effective tumor immunotherapy. Expert Rev. Vaccines.

[102] Sznol M., Chen L. (2013). Antagonist antibodies to PD-1 and B7-H1 (PD-L1) in the treatment of advanced human cancer. Clin. Cancer Res..

[103] Garnett-Benson C., Hodge J.W., Gameiro S.R. (2015). Combination regimens of radiation therapy and therapeutic cancer vaccines: Mechanisms and opportunities. Semin. Radiat. Oncol..

[104] Gameiro S.R., Jammeh M.L., Wattenberg M.M., Tsang K.Y., Ferrone S., Hodge J.W. (2014). Radiation-induced immunogenic modulation of tumor enhances antigen processing and calreticulin exposure, resulting in enhanced T-cell killing. Oncotarget.

[105] Xie N., Shen G., Gao W., Huang Z., Huang C., Fu L. (2023). Neoantigens: Promising targets for cancer therapy. Signal Transduct. Target. Ther..

[106] Vohra J., Barbosa G.R., Reis L.O. (2025). mRNA and DNA-Based Vaccines in Genitourinary Cancers: A New Frontier in Personalized Immunotherapy. Vaccines.

[107] Garcia-Garijo A., Fajardo C.A., Gros A. (2019). Determinants for Neoantigen Identification. Front. Immunol..

[108] Bahlinger V., Hartmann A., Eckstein M. (2023). Immunotherapy in Genitourinary Cancers: Role of Surgical Pathologist for Detection of Immunooncologic Predictive Factors. Adv. Anat. Pathol..

[109] Zhang X., Qi Y., Zhang Q., Liu W. (2019). Application of mass spectrometry-based MHC immunopeptidome profiling in neoantigen identification for tumor immunotherapy. BioMed. Pharmacother..

[110] Cai Y., Gong M., Zeng M., Leng F., Lv D., Guo J., Wang H., Li Y., Lin Q., Jing J. (2025). Immunopeptidomics-guided discovery and characterization of neoantigens for personalized cancer immunotherapy. Sci. Adv..

[111] Reustle A., Di Marco M., Meyerhoff C., Nelde A., Walz J.S., Winter S., Kandabarau S., Büttner F., Haag M., Backert L. (2020). Integrative -omics and HLA-ligandomics analysis to identify novel drug targets for ccRCC immunotherapy. Genome Med..

[112] Huber F., Arnaud M., Stevenson B.J., Michaux J., Benedetti F., Thevenet J., Bobisse S., Chiffelle J., Gehert T., Müller M. (2025). A comprehensive proteogenomic pipeline for neoantigen discovery to advance personalized cancer immunotherapy. Nat. Biotechnol..

[113] Scheid J., Lemke S., Hoenisch-Gravel N., Dengler A., Sachsenberg T., Declerq A., Gabriels R., Bauer J., Wacker M., Bichmann L. (2025). MHCquant2 refines immunopeptidomics tumor antigen discovery. Genome Biol..

[114] Wu A.C., Nakamura Y., Kiyotani K. (2025). Advances in Neoantigen-Based Cancer Vaccines. Cancers.

[115] Ott P.A., Hu Z., Keskin D.B., Shukla S.A., Sun J., Bozym D.J., Zhang W., Luoma A., Giobbie-Hurder A., Peter L. (2017). An immunogenic personal neoantigen vaccine for patients with melanoma. Nature.

[116] Sahin U., Derhovanessian E., Miller M., Kloke B.-P., Simon P., Löwer M., Bukur V., Tadmor A.D., Luxemburger U., Schrörs B. (2017). Personalized RNA mutanome vaccines mobilize poly-specific therapeutic immunity against cancer. Nature.

[117] Flores-Islas A.K., Rico-Fuentes C., Sierra-Díaz E., García-Chagollán M., Pereira-Suárez A.L., Zepeda-Nuño J.S., Moreno-Ortiz J.M., Ramírez-De-Arellano A. (2026). Immunotherapeutic Strategies for Prostate Cancer: A Comprehensive Review. Cancers.

[118] Bou-Dargham M.J., Sha L., Sang Q.A., Zhang J. (2020). Immune landscape of human prostate cancer: Immune evasion mechanisms and biomarkers for personalized immunotherapy. BMC Cancer.

[119] Lv Y.-H., He Y.-C., Dai X.-Y., Yang X.-J., Cai Y.-S., Luo R.-H., Xie Q.-Y., Xie S.-N., Chen X.-T., Zhou Q.-B. (2025). Alternative splicing: From tumorigenesis to neoantigen-mediated cancer immunotherapy. Biomark. Res..

[120] Peng K., Zhao X., Fu Y.X., Liang Y. (2025). Eliciting antitumor immunity via therapeutic cancer vaccines. Cell. Mol. Immunol..

[121] Baljon J.J., Kwiatkowski A.J., Pagendarm H.M., Stone P.T., Kumar A., Bharti V., Schulman J.A., Becker K.W., Roth E.W., Christov P.P. (2024). A Cancer Nanovaccine for Co-Delivery of Peptide Neoantigens and Optimized Combinations of STING and TLR4 Agonists. ACS Nano.

[122] Fan Y., Moon J.J. (2015). Nanoparticle Drug Delivery Systems Designed to Improve Cancer Vaccines and Immunotherapy. Vaccines.

[123] Wells K., Liu T., Zhu L., Yang L. (2024). Immunomodulatory nanoparticles activate cytotoxic T cells for enhancement of the effect of cancer immunotherapy. Nanoscale.

[124] Lei J., Qi S., Yu X., Gao X., Yang K., Zhang X., Cheng M., Bai B., Feng Y., Lu M. (2024). Development of Mannosylated Lipid Nanoparticles for mRNA Cancer Vaccine with High Antigen Presentation Efficiency and Immunomodulatory Capability. Angew. Chem. Int. Ed. Engl..

[125] Kong W., Wei Y., Dong Z., Liu W., Zhao J., Huang Y., Yang J., Wu W., He H., Qi J. (2024). Role of size, surface charge, and PEGylated lipids of lipid nanoparticles (LNPs) on intramuscular delivery of mRNA. J. Nanobiotechnol..

[126] Chilumula S., Hanchate P., Patri S.V., Marepally S. (2025). Influence of structural modifications in synthetic vectors of lipid adjuvants on mRNA vaccine delivery. Biomater. Sci..

[127] Quan Y., Yang H., Li W., Li L. (2025). mRNA vaccines: Immunogenicity and quality characteristics. J. Nanobiotechnol..

[128] Du J., Fan Z., Huang J., Li Z., Hu H., Li Y. (2025). The rise of mRNA therapeutic vaccines. RSC Pharm..

[129] Wang J., Ding Y., Chong K., Cui M., Cao Z., Tang C., Tian Z., Hu Y., Zhao Y., Jiang S. (2024). Recent Advances in Lipid Nanoparticles and Their Safety Concerns for mRNA Delivery. Vaccines.

[130] Tenchov R., Bird R., Curtze A.E., Zhou Q. (2021). Lipid Nanoparticles—From Liposomes to mRNA Vaccine Delivery, a Landscape of Research Diversity and Advancement. ACS Nano.

[131] Rueda F., Eich C., Cordobilla B., Domingo P., Acosta G., Albericio F., Cruz L.J., Domingo J.C. (2017). Effect of TLR ligands co-encapsulated with multiepitopic antigen in nanoliposomes targeted to human DCs via Fc receptor for cancer vaccines. Immunobiology.

[132] Lung P., Yang J., Li Q. (2020). Nanoparticle formulated vaccines: Opportunities and challenges. Nanoscale.

[133] Zhang Y., Li Y., Chen K., Qian L., Wang P. (2021). Oncolytic virotherapy reverses the immunosuppressive tumor microenvironment and its potential in combination with immunotherapy. Cancer Cell Int..

[134] Cao G., Ding C., Dai J., Qiu X. (2025). Oncolytic virus and immunogenic cell death in cancer therapy. Tumour Virus Res..

[135] Rivera-Orellana S., Bautista J., Palacios-Zavala D., Ojeda-Mosquera S., Altamirano-Colina A., Alcocer-Veintimilla M., Parrales-Rosales G., Izquierdo-Condoy J.S., Vásconez-González J., Ortiz-Prado E. (2025). Oncolytic virotherapy and tumor microenvironment modulation. Clin. Exp. Med..

[136] Blee C., Muthana M., Wells G., Danson S. (2025). Turning the tide: Harnessing vaccines and viruses to fight cancer. Immunother. Adv..

[137] Joffre O.P., Segura E., Savina A., Amigorena S. (2012). Cross-presentation by dendritic cells. Nat. Rev. Immunol..

[138] Melief C.J., van Hall T., Arens R., Ossendorp F., van der Burg S.H. (2015). Therapeutic cancer vaccines. J. Clin. Investig..

[139] Page S.T., Plymate S.R., Bremner W.J., Matsumoto A.M., Hess D.L., Lin D.W., Amory J.K., Nelson P.S., Wu J.D. (2006). Effect of medical castration on CD4^+^ CD25^+^ T cells, CD8^+^ T cell IFN-gamma expression, and NK cells: A physiological role for testosterone and/or its metabolites. Am. J. Physiol. Endocrinol. Metab..

[140] Drake C.G., Doody A.D., Mihalyo M.A., Huang C.-T., Kelleher E., Ravi S., Hipkiss E.L., Flies D.B., Kennedy E.P., Long M. (2005). Androgen ablation mitigates tolerance to a prostate/prostate cancer-restricted antigen. Cancer Cell.

[141] Formenti S.C., Demaria S. (2009). Systemic effects of local radiotherapy. Lancet Oncol..

[142] Golden E.B., Frances D., Pellicciotta I., Demaria S., Helen Barcellos-Hoff M., Formenti S.C. (2014). Radiation fosters dose-dependent and chemotherapy-induced immunogenic cell death. Oncoimmunology.

[143] Vanpouille-Box C., Alard A., Aryankalayil M.J., Sarfraz Y., Diamond J.M., Schneider R.J., Inghirami G., Coleman C.N., Formenti S.C., Demaria S. (2017). DNA exonuclease Trex1 regulates radiotherapy-induced tumour immunogenicity. Nat. Commun..

[144] Yang M., Cui M., Sun Y., Liu S., Jiang W. (2024). Mechanisms, combination therapy, and biomarkers in cancer immunotherapy resistance. Cell Commun. Signal..

[145] Wang J., Guo R., Zhang L., Zuo W., Li X., Zhang S., Tan Q., Ma J. (2025). Reversing the “cold” tumor microenvironment: The role of neoantigen vaccines in prostate cancer. J. Transl. Med..

[146] Walter S., Weinschenk T., Stenzl A., Zdrojowy R., Pluzanska A., Szczylik C., Staehler M., Brugger W., Dietrich P.-Y., Mendrzyk R. (2012). Multipeptide immune response to cancer vaccine IMA901 after single-dose cyclophosphamide associates with longer patient survival. Nat. Med..

[147] Melero I., Gaudernack G., Gerritsen W., Huber C., Parmiani G., Scholl S., Thatcher N., Wagstaff J., Zielinski C., Faulkner I. (2014). Therapeutic vaccines for cancer: An overview of clinical trials. Nat. Rev. Clin. Oncol..

[148] Chan T., Yarchoan M., Jaffee E., Swanton C., Quezada S., Stenzinger A., Peters S. (2019). Development of tumor mutation burden as an immunotherapy biomarker: Utility for the oncology clinic. Ann. Oncol..

[149] Rizvi N.A., Hellmann M.D., Snyder A., Kvistborg P., Makarov V., Havel J.J., Lee W., Yuan J., Wong P., Ho T.S. (2015). Cancer immunology. Mutational landscape determines sensitivity to PD-1 blockade in non-small cell lung cancer. Science.

[150] Rosenberg J.E., Hoffman-Censits J., Powles T., van der Heijden M.S., Balar A.V., Necchi A., Dawson N., O’Donnell P.H., Balmanoukian A., Loriot Y. (2016). Atezolizumab in patients with locally advanced and metastatic urothelial carcinoma who have progressed following treatment with platinum-based chemotherapy: A single-arm, multicentre, phase 2 trial. Lancet.

[151] Alexandrov L.B., Kim J., Haradhvala N.J., Huang M.N., Ng A.W.T., Wu Y., Boot A., Covington K.R., Gordenin D.A., Bergstrom E.N. (2020). The repertoire of mutational signatures in human cancer. Nature.

[152] Slovin S.F. (2016). Biomarkers for immunotherapy in genitourinary malignancies. Urol. Oncol..

[153] Bassani-Sternberg M., Bräunlein E., Klar R., Engleitner T., Sinitcyn P., Audehm S., Straub M., Weber J., Slotta-Huspenina J., Specht K. (2016). Direct identification of clinically relevant neoepitopes presented on native human melanoma tissue by mass spectrometry. Nat. Commun..

[154] Braun D.A., Hou Y., Bakouny Z., Ficial M., Angelo M.S., Forman J., Ross-Macdonald P., Berger A.C., Jegede O.A., Elagina L. (2020). Interplay of somatic alterations and immune infiltration modulates response to PD-1 blockade in advanced clear cell renal cell carcinoma. Nat. Med..

[155] Lu L., Lu X., Luo W. (2025). Personalized Cancer Vaccines: Current Advances and Emerging Horizons. Vaccines.

[156] Ho S.-Y., Chang C.-M., Liao H.-N., Chou W.-H., Guo C.-L., Yen Y., Nakamura Y., Chang W.-C. (2023). Current Trends in Neoantigen-Based Cancer Vaccines. Pharmaceuticals.

[157] Li L., Goedegebuure S.P., Gillanders W.E. (2017). Preclinical and clinical development of neoantigen vaccines. Ann. Oncol..

[158] Li X., You J., Hong L., Liu W., Guo P., Hao X. (2023). Neoantigen cancer vaccines: A new star on the horizon. Cancer Biol. Med..

[159] Hao Q., Long Y., Yang Y., Deng Y., Ding Z., Yang L., Shu Y., Xu H. (2024). Development and Clinical Applications of Therapeutic Cancer Vaccines with Individualized and Shared Neoantigens. Vaccines.

[160] Murphy J.F. (2025). Personalized Vaccines: Unlocking the Next Era of Medical Innovation in Cancer Immunotherapy. Cancer Screen. Prev..

[161] Saade F., Petrovsky N. (2012). Technologies for enhanced efficacy of DNA vaccines. Expert Rev. Vaccines.

[162] Maiorano B.A., Schinzari G., Ciardiello D., Rodriquenz M.G., Cisternino A., Tortora G., Maiello E. (2021). Cancer Vaccines for Genitourinary Tumors: Recent Progresses and Future Possibilities. Vaccines.

